# Silencing of VEGFR2 by RGD-Modified Lipid Nanoparticles Enhanced the Efficacy of Anti-PD-1 Antibody by Accelerating Vascular Normalization and Infiltration of T Cells in Tumors

**DOI:** 10.3390/cancers12123630

**Published:** 2020-12-04

**Authors:** Riki Cho, Yu Sakurai, Haleigh Sakura Jones, Hidetaka Akita, Akihiro Hisaka, Hiroto Hatakeyama

**Affiliations:** 1 Laboratory of Clinical Pharmacology and Pharmacometrics, Graduate School of Pharmaceutical Sciences, Chiba University, 1-8-1 Inohana, Chuo-ku, Chiba, Chiba 260-8675, Japan; aeka1715@chiba-u.jp (R.C.); sjones@chiba-u.jp (H.S.J.); hisaka@chiba-u.jp (A.H.); 2 Laboratory of Pharmacology and Toxicology, Graduate School of Pharmaceutical Sciences, Chiba University, 1-8-1 Inohana, Chuo-ku, Chiba, Chiba 260-8675, Japan; yu_sakurai@chiba-u.jp (Y.S.); akitahide@chiba-u.jp (H.A.)

**Keywords:** aPD-1, ssPalm, RGD, immune checkpoint inhibitor, siRNA, vascular normalization, lipid nanoparticle

## Abstract

**Simple Summary:**

siRNA delivery to tumor endothelial cells was achieved using arginyl-glycyl-aspartic acid (RGD)-modified lipid nanoparticles containing a novel pH-sensitive and biodegradable lipid. The anti-tumor efficacy of an immune checkpoint inhibitor was improved by the silencing of VEGFR2 using the delivery system, because the combination therapy induced vascular normalization and increased CD8+ T cell infiltration into tumors. The efficient delivery of nucleic acids is a promising strategy to improve therapeutic outcomes in immune checkpoint inhibitor-resistant cancers.

**Abstract:**

Despite the promising anticancer effects of immune checkpoint inhibitors, their low objective response rate remains to be resolved; thus, combination therapies have been investigated. We investigated the combination of an anti-programmed cell death 1 (aPD-1) monoclonal antibody with the knockdown of vascular endothelial factor receptor 2 (VEGFR2) on tumor endothelial cells to overcome resistance to immune checkpoint inhibitors and improve the objective response rate. The successful delivery of small interfering RNA to tumor endothelial cells was achieved by RGD peptide-modified lipid nanoparticles composed of a novel, pH-sensitive, and biodegradable ssPalmO-Phe. RGD-modified lipid nanoparticles efficiently induced the knockdown of VEGFR2 in tumor endothelial cells (TECs), which induced vascular normalization. The combination of a PD-1 monoclonal antibody with Vegfr2 knockdown enhanced CD8+ T cell infiltration into tumors and successfully suppressed tumor growth and improved response rate compared with monotherapy. Our combination approach provides a promising strategy to improve therapeutic outcomes in immune checkpoint inhibitor-resistant cancers.

## 1. Introduction

Immune checkpoint inhibitors (ICIs) that target programmed cell death-1 (PD-1) or programmed cell death-ligand 1 (PD-L1) proteins are being used extensively, for the treatment of many types of cancers. Anti-PD-1/PD-L1 monoclonal antibodies (mAbs) have been used as monotherapies and are associated with low objective response rates (ORRs) and acquired tumor resistance [1,2]. Thus, there is a need for combination therapies. Clinical trials have been conducted to investigate the synergistic effect of ICIs and anti-angiogenesis in patients [3,4].

Angiogenesis, the generation of new blood vessels from preexisting vessels, occurs in many physiological processes, including tumorigenesis by pro-angiogenic factors [5]. The vessel maturation process is impeded because of the persistent hypersecretion of pro-angiogenic factors in the tumor microenvironment [6]. Abnormal angiogenesis leads to a lack of pericyte coverage and leaky blood vessels, resulting in increased vascular permeability and higher interstitial fluid pressure, which inhibits the infiltration of cytotoxic lymphocytes into tumors [7,8]. Immature blood vessels cannot supply enough oxygen to compensate for consumption, thus resulting in hypoxia, which directly impairs the functions of tumor infiltrating lymphocytes (TILs).

Vascular endothelial growth factor (VEGF) plays a pivotal role in angiogenesis in tumor microenvironments [9,10]. Bevacizumab, an anti-VEGF mAb, is the first anti-angiogenic agent approved for multiple cancers [11]. However, the neutralization of VEGF results in systemic side effects, including hemorrhage, hypertension, proteinuria, impaired wound healing, and thrombosis [12]. Therefore, angiogenesis factors must be selectively targeted in the tumor microenvironment. The angiogenic signal of VEGF is mainly transmitted by its receptor, VEGFR2, which is expressed on tumor endothelial cells (TECs) [10]. We previously developed arginyl-glycyl-aspartic acid (RGD)-modified liposomal delivery systems targeting TECs [13,14,15,16,17]. RGD-modified liposomes were used to deliver doxorubicin to TECs, killing these cells and destroying the tumor vessel structure in doxorubicin-resistant renal cell carcinoma, while the blood vessels in normal organs such as the liver and spleen were not affected by this drug delivery system [13]. This research showed that RGD-modified liposomes selectively deliver doxorubicin to TECs. We also demonstrated that small interfering RNAs (siRNA) against Vegfr2 delivered to TECs in murine tumors by RGD-modified lipid nanoparticles (LNPs) resulted in vascular normalization [15,17].

We reported the development of a series of ionizable lipids, an SS-cleavable and pH-activated lipid-like material (ssPalm), as a material for use as a nucleic acid carrier [18,19,20]. Recently, we synthesized a novel ssPalmO-Phenyl-P4C2 (ssPalmO-Phe) that was characterized by self-degradability driven by redox-responsive concentrations of the thiol group and subsequent nucleophilic attacks of thiol groups against phenyl esters [21]. This spontaneous degradation inside the cells allows its cargo to be effectively released into the cytosol, thus achieving efficient delivery of mRNA. In the study reported here, we demonstrated the improved effect of an anti-programmed cell death 1 (aPD-1) mAb with vascular normalization caused by the knockdown of Vegfr2, by delivering siRNA using RGD-modified LNP composed of ssPalmO-Phe.

## 2. Results

### 2.1. Characterization of LNPs

LNPs were prepared using ethanol dilution, and the PEG-lipid or RGD-PEG-lipid was modified using the post-insertion method, as described in the Materials and Methods section. The average diameters of the prepared PEG-LNP and RGD-LNP comprising ssPalmO-Phe were approximately 160 nm, and they possessed a slightly negative charge (Table 1); this indicates that RGD modification did not affect the physicochemical characteristics of LNPs. The polydispersity index (PDI) of the two LNPs, as an indicator of homogeneity of particle diameter distribution, was approximately 0.2. The value meant that PEG-LNP and RGD-LNP were homogeneous nanoparticles. siRNA in LNPs had an encapsulation efficiency of more than 90%, comparable to that of LNPs composed of other types of ssPalm [18]. The recovery rate of each LNP suggests that approximately half of the starting amount of siRNA was encapsulated in LNP, which is also consistent with our previous data [18]. The siRNAs were successfully encapsulated in LNPs prepared with ssPalmO-Phe.

### 2.2. In Vitro Cellular Uptake and Knockdown by LNPs in Endothelial Cells

**Figure 1:**
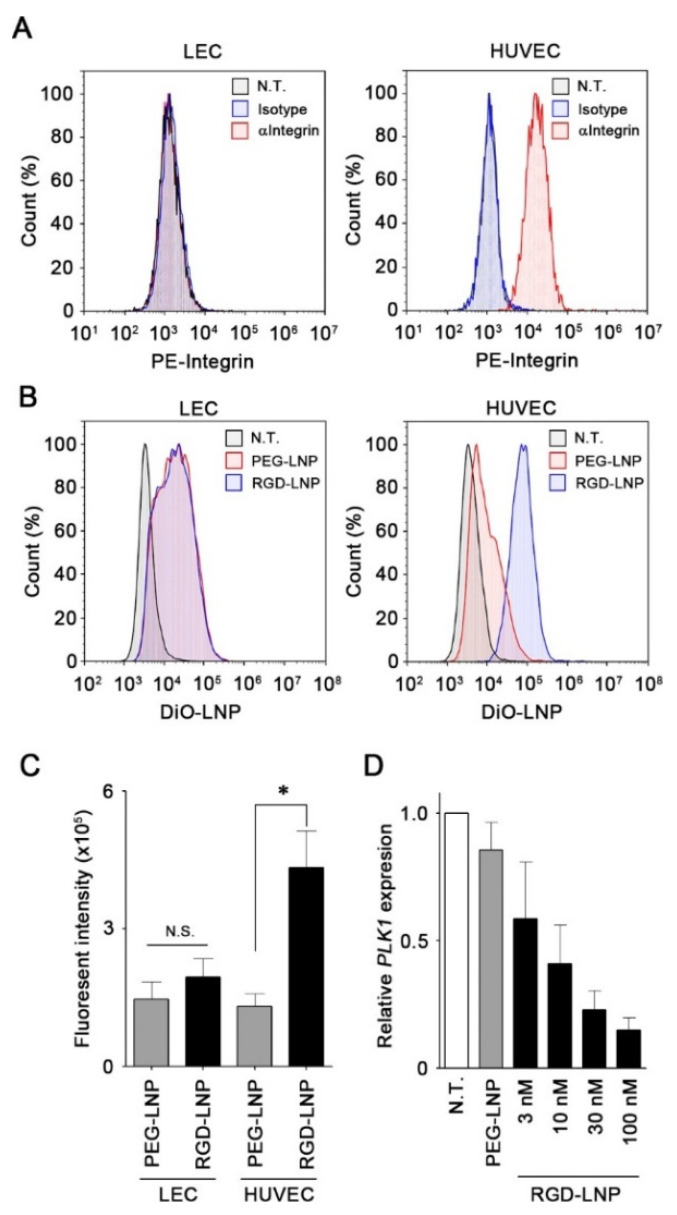
In vitro *PLK1* knockdown by RGD-LNP in human endothelial cells expressing integrin. (**A**) Representative histograms based on flow cytometric analysis for LEC and HUVEC stained with the control antibody or anti-integrin antibody. (**B**) Representative histograms based on flow cytometric analysis of the uptake of PEG-LNP or RGD-LNP labeled with DiO in LEC or HUVEC. (**C**) Cellular uptake of PEG-LNP or RGD-LNP labeled with DiO in LEC or HUVEC measured using flow cytometric analysis. Data are shown as the mean ± S.E. (*n* = 3). * *p* < 0.05. (**D**) Expression of *PKL1* in HUVEC after treatment with PEG-LNP at 100 nM or RGD-LNP at indicated concentrations of siPLK1 for 24 h. Data are shown as the mean ± S.E. (*n* = 3). N.T.: No treatment.

To evaluate the expression of the integrin receptors on the cell surface, lymphatic endothelial cells (LEC) and Human umbilical vein endothelial cells (HUVEC) were analyzed using flow cytometry. HUVEC cells expressed substantial levels of integrin; however, no expression of integrin was detected in the LEC cells (Figure 1A). We next examined the cellular uptake of PEG-LNP and RGD-LNP by LEC and HUVEC cells. Although no difference in uptake of LNPs was detected in LEC cells, a significant increase in cellular uptake was observed for RGD-LNP in HUVEC cells (Figure 1B,C). These results demonstrated that RGD modification selectively enhanced the cellular uptake of LNPs in integrin-positive cells such as HUVEC cells. The knockdown efficacy of RGD-LNP and RGD-LNP in HUVEC cells was examined using RT-PCR, which revealed that PEG-LNP showed negligible knockdown of *PLK1* in HUVEC cells at a dose of 100 nM of siRNA, compared with untreated HUVEC. However, RGD-LNP decreased the expression of *PLK1* in a dose-dependent manner (Figure 1D). This finding clearly demonstrated that RGD-LNP could induce the knockdown of target genes by delivering siRNA selectively to integrin-positive endothelial cells.

### 2.3. In Vivo Knockdown of VEGFR2 on TECs and Vascular Normalization

**Figure 2:**
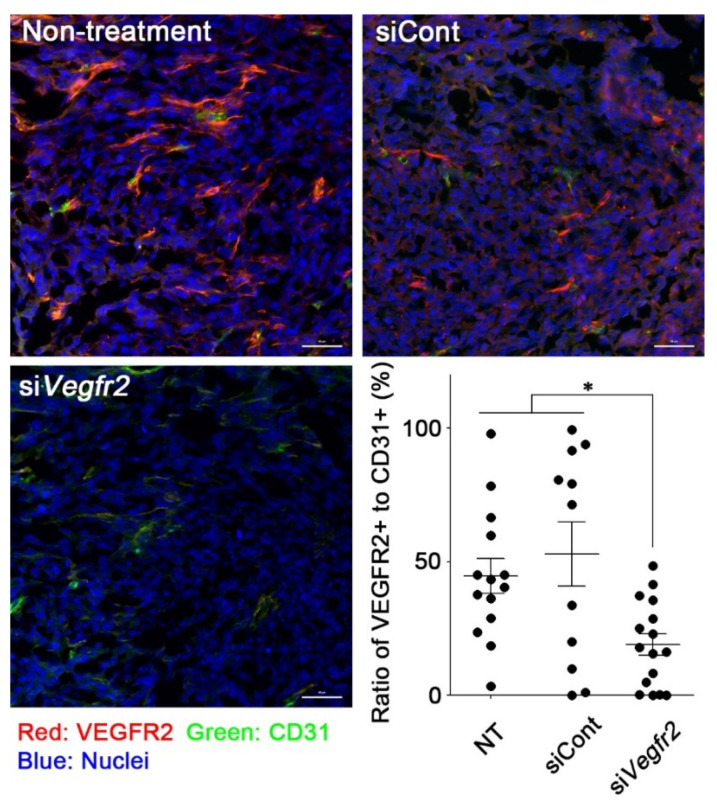
In vivo *Vegfr2* knockdown in MC38 tumors by delivery of si*Vegfr2* using RGD-LNP. MC38 tumor-bearing mice were administered with RGD-LNP encapsulating either si*PLK1* as a control, or si*Vegfr2*, at a dose of 40 μg siRNA. Tumors were harvested 24 h after administration, sectioned, and stained as described in the Materials and Methods section. Four tumors from each group were imaged, and CD31-positive cells stained with VEGFR2 or not stained were quantified in each image. Red: VEGFR2, green: CD31 (marker of endothelial cells), blue: nuclei. * *p* < 0.05 determined using one-way ANOVA followed by Tukey’s test. Bars: 50 μm.

**Figure 3:**
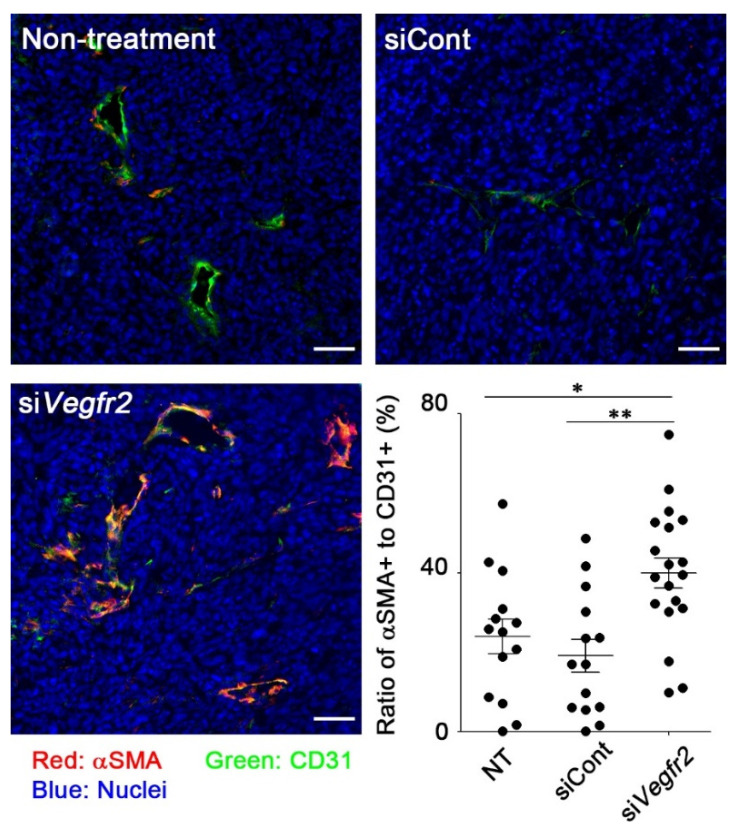
Vascular normalization in MC38 tumors by knockdown of *Vegfr2*. MC38 tumor-bearing mice were administered with RGD-LNP encapsulating either si*PLK1* as a control, or si*Vegfr2*, at a dose of 40 μg siRNA twice, three days apart. Tumors were harvested 24 h after the final administration, sectioned, and stained as described in the Materials and Methods section. Four tumors from each group were imaged, and CD31-positive cells that were stained with αSAM or not stained were quantified in each image. Red: αSMA (marker of pericytes), green: CD31 (marker of endothelial cells), blue: nuclei. * *p* < 0.05, ** *p* < 0.01 determined using one-way ANOVA followed by Tukey’s test. Bars: 50 μm.

To evaluate whether RGD-LNP could induce the knockdown of a target gene in murine TECs, RGD-LNP encapsulating si*Vegfr2* was intravenously administered to MC38 tumor-bearing mice. To distinguish endothelial cells from other cells, CD31, a marker of endothelial cells, was also stained as described in the Materials and Methods section. VEGFR2 expression on CD31-positive cells was significantly decreased after treatment with RGD-LNP encapsulating si*Vegfr2* (Figure 2). Vascular normalization was initiated in tumors treated with RGD-LNP encapsulating si*Vegfr2*. The marge ratio of αSMA, a marker of pericytes, in positive TECs with CD31 in tumors treated with control siRNA was comparable to that in untreated tumors (Figure 3). An increase in the ratio of αSMA to CD31 was, however, observed in tumors treated with si*Vegfr2*. The results clearly showed that knockdown of *Vegfr2* promoted coverage of tumor vasculature with pericytes, i.e., vascular normalization. When used in combination, RGD-LNP successfully delivered si*Vegfr2* to TECs in MC38 tumors.

### 2.4. In Vivo Anti-Tumor Efficacy

**Figure 4:**
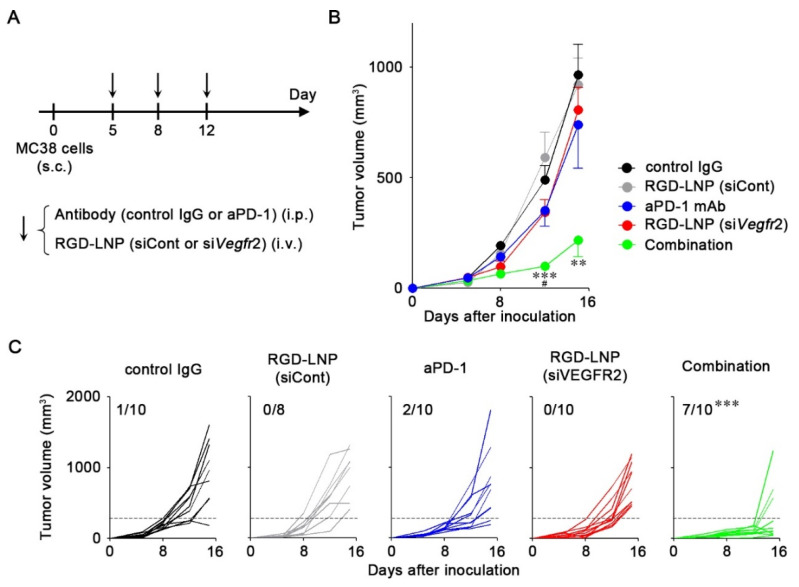
Combination therapy involving aPD-1 and Vegfr2 knockdown. (**A**) Experimental schedule of treatment of MC38 tumor-bearing mice with aPD-1 and RGD-LNP. On day 5, 8, and 12 after tumor inoculation, antibodies (control IgG or aPD-1) at 200 μg/mouse and RGD-LNP (siPLK1 as a control or siVegfr2) at 40 μg siRNA/mouse were intraperitoneally (i.p.) and intravenously (i.v.) administered. (**B**) The average tumor volume of MC38 tumor-bearing mice is shown as the mean ± S.E. (*n* = 8–10). ** *p* < 0.01 and *** *p* < 0.001 vs. control IgG and siCont, ^#^ *p* < 0.05 combination therapy vs. monotherapies (aPD-1 and RGD-LNP (siVegfr2)) determined using one-way ANOVA followed by Tukey’s test. (**C**) Changes in tumor volume for MC38. The numbers show tumors with <30% of the average volume of tumors treated with control IgG on the last day (dotted lines). *** *p* < 0.0001 combination vs. other groups determined using chi-squared test followed by adjusted standardized residual analysis.

**Figure 5:**
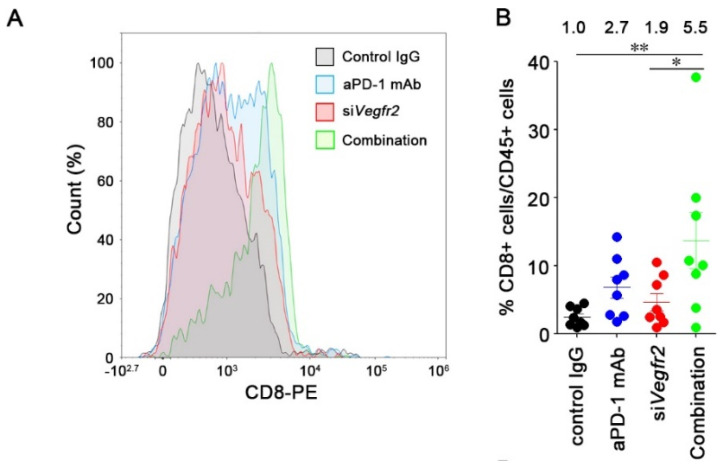

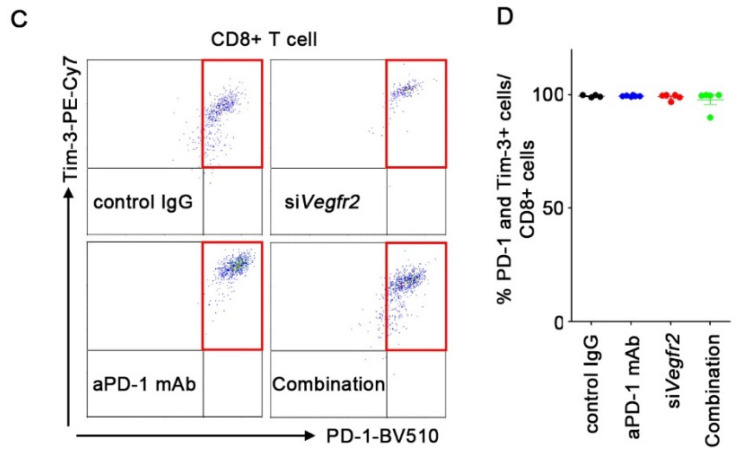
Effect of combination therapy on T cell infiltrations in MC38 tumors. MC38 tumor-bearing mice were treated on days 5, 8, and 12, and tumors were harvested on day 13 for flow cytometric analysis. (**A**) Representative flow plots of CD8+ T cells from MC38 tumors. (**B**) Frequency of CD8+ T cells (CD45+CD3+CD8+) in CD45+ cells in MC38 tumors. Mean ± S.E. (*n* = 8). * *p* < 0.05 and ** *p* < 0.01 determined using one-way ANOVA followed by Tukey’s test. (**C**) Representative flow plots of PD-1 and Tim-3 fluorescence intensities on isolated CD8+ T cells from MC38 tumors. (**D**) Frequency of PD-1+Tim-3+ cells in CD8+ T cells in MC38 tumors. Mean ± S.E. (*n* = 6).

We examined the anti-tumor efficacy of aPD-1 mAb combined with *Vegfr2* knockdown (Figure 4A). Monotherapy with either aPD-1 mAb or RGD-LNP had a minor effect on MC38 tumor growth (Figure 4B). Combination therapy significantly suppressed tumor growth compared with controls and monotherapies by day 12. We also assessed the interaction index of the combination therapy versus monotherapy [22]. The interaction indexes of the combination therapy at days 12 and 15 were −0.90 (95% confidence interval (CI), −1.55 to −0.26) and −0.64 (95% CI, −1.56 to 0.29), respectively, which indicates that the combination of aPD-1 and siVegfr2 was additive to supra-additive as compared with the monotherapies. The combination therapy also induced strong responses in seven of ten tumor-bearing mice (Figure 4C). Significant differences were observed in the response rate of the combination therapy compared with those of other groups. Anti-angiogenesis caused by the silencing of *Vegfr2* improved the response to an immune checkpoint inhibitor. Thereafter, we investigated the mechanisms by which the combination successfully suppressed tumor growth by analyzing CD8+ T cells that infiltrated the tumors. When compared with control IgG treatment, aPD-1 mAb or si*Vegfr2* treatment resulted in a 2.7- and 1.9-fold increase in infiltrated CD8+ T cells in tumors (Figure 5A,B). The infiltration of CD8+ T cells increased 5.5-fold after the combination therapy. We further analyzed the exhausted markers such as PD-1 and T-cell immunoglobulin mucin-3 (Tim-3) on CD8+ T cells by isolating CD8+ T cells from tumors. No changes in the expression of these markers were found due to the treatments (Figure 5C,D), which suggested that the unresponsiveness of CD8+ T cells to aPD-1 mAb in MC38 was not caused by the exhaustion state. The improved anti-tumor efficacy of aPD-1 mAb combined with the knockdown of *Vegfr2* resulted from the increase in infiltration of CD8+ T cells in tumors, but not from the prevention of CD8+ T cell exhaustion.

## 3. Discussion

The objective response rate (ORR) to aPD-1 mAbs such as nivolumab and pembrolizumab has been reported to be limited to around 20% [2]. Cytotoxic T-lymphocyte-associated protein 4 (CTLA-4) and PD-1 blockade have proven successful in improving survival rates, resulting in the approval of the ipilimumab and nivolumab combination for the treatment of metastatic melanoma and renal cell carcinoma [23]. However, severe side effects have been observed with combination therapy. The development of safer alternative means is necessary.

Tumors with high levels of TIL respond better to ICIs than tumors with low TIL [1]. Therefore, strategies that can convert “cold” tumors into “hot” tumors have attracted attention, as they improve the ORR of ICIs. One promising approach involves combining ICIs with anti-angiogenic agents, because pro-angiogenic factors can play both direct and indirect roles in the immunosuppressive tumor microenvironment [4]. Several studies have shown that treatment of xenograft cancer models with inhibitors of VEGF or VEGFR2, such as anti-VEGFR2 receptor antibodies and apatinib, can exert a synergistic effect with aPD-1 or aPD-L1 mAbs [24,25,26,27,28,29,30]. Anti-PD-L1 atezolizumab in combination with bevacizumab has been shown to enhance the migration of TILs in patients with metastatic renal cell carcinoma [31]. Clinical trials combining immunotherapy and anti-angiogenic factors such as apatinib, lenvatinib, and ramucirumab are currently in progress for several types of cancer, suggesting that combining a strategy with anti-angiogenesis is beneficial for immunotherapy [32,33,34,35].

We found that the knockdown of *Vegfr2* in TECs enhanced the anti-tumor efficacy of aPD-1 mAb. Selective targeting of TECs was achieved by RGD-modified LNP, because RGD modification alters the biodistribution of nanocarrier systems, facilitating their delivery to integrin αvβ3-expresisng TECs [36,37,38]. The size of LNPs which target cancer cells in tumors is generally less than 100 nm in diameter, because LNPs have to pass through the gap between the TECs of the neovasculature, based on the enhanced permeability and retention (EPR) effect [39]. In this study, the diameter of the prepared LNPs was 160 nm, which is appropriate for targeting TECs of the neovasculature due to a reduction in the EPR effect [13,40]. LNPs composed of ssPalmO-Phe showed lower toxicity because of their biodegradability [21]. Biodegradable LNPs deliver nucleic acids in vivo without any apparent toxicity. In a preclinical safety study, no significant changes in the biochemical parameters of blood, such as the number of red blood cells, hematocrit, mean corpuscular volume, number of platelets, or the number of white blood cells, was observed, even when LNPs composed of ssPalmO-Phe were administered to rats at a dose of 175 mg nucleic acids/kg body weight [21]. This delivery system can be used for delivering not only siRNA but also other nucleic acids such as mRNA to TECs, to efficiently manipulate undruggable angiogenesis factors that cannot be inhibited by small compounds or antibodies.

It was demonstrated that the efficacy of ICIs could be improved in combination with the knockdown of *Vegfr2* in TECs. Although treatment with either aPD-1 mAb or si*Vegfr2* enhanced tumor infiltrating CD8+ T cells in tumors by two to three-fold compared with control treatment, the combination further increased the infiltration of CD8+ T cells into tumors by 5.5-fold. Type 1 T helper (Th1) cells, such as CD8+ T cells in tumors, promote vascular normalization by secreting interferon-γ (IFN-γ) [41]. T cells that infiltrate tumors after vascular normalization were activated by aPD-1 mAbs and could secrete IFN-γ to promote further vascular normalization, which could explain the increase in CD8+ T cells after the combination treatment. Vascular normalization in tumors also enhanced the delivery of antibody and protein therapeutics to tumors [42,43]. Therefore, vascular normalization induced by the knockdown of *Vegfr2* could contribute to an increase in the amount of aPD-1 mAb delivered in tumors. When used in combination, a blockade of both pathways efficiently converts the cold tumor microenvironment into a hot microenvironment. As previously reported, the enhanced anti-tumor effect of ICIs combined with anti-angiogenesis was observed in subcutaneous xenograft models [24,25,28]. However, subcutaneous xenograft tumors do not necessarily reflect the actual tumor microenvironment. The synergistic effect between ICIs and anti-angiogenesis has been also observed in orthotopic models [26,29,30]. Even though further study is required to evaluate the delivery of LNPs in orthotopic models of colon cancer in terms of the delivery mechanisms of LNPs in the peritoneal compartment and the efficacy of their cargos, the combination of PD-1/PD-L1 blockade with si*Vegfr2* delivery by the LNP system could be applicable for the treatment of peritoneal disseminations.

In some cases, T cells are ineffective against cancer because the T cells enter exhaustion, a state of T cell dysfunction [44]. It was reported that anti-PD-1/PD-L1 upregulates the Ras-Raf-MEK-ERK and PI3K-AKT signaling pathways in immune cells by blocking the PD-1/PD-L1 axis [45]. Anti-PD-1/PD-L1 therapy therefore restores T cells from an exhausted state and enhances their tumor-killing activity [3]. In the present study, a reduction in the expression of the markers of exhaustion, PD-1 and Tim-3, was not observed, even after the combination therapy. Even though further studies are required to elucidate the precise mechanisms of action, T cell exhaustion in tumors is not a dominant factor for the immune surveillance of MC38 tumors.

## 4. Materials and Methods 

### 4.1. Materials 

Murine colon adenocarcinoma MC38 cells were purchased from Kerafast (Boston, MA, USA). Human umbilical vein endothelial cells (HUVEC) and lymphatic endothelial cells (LEC) were obtained from LONZA (Basel, Switzerland). Anti-PD-1 mAb (RMP1-14) and IgG2a isotype control mAb (2A3) were purchased from Bioxcell (West Lebanon, NH, USA). Anti-mouse *Vegfr2* siRNA (sense: 5′-cAA ccA GAG Acc cuc Guu udTsdT-3′, antisense: 5′-AAA CGA GGG UCU CUG GUU GdTsdT-3′) as previously reported [46], and anti-human *PLK1* siRNA (sense: 5′-AGA uCA CCC uCC UuA AAu AUU-3′, antisense: 5′-UAU UUA AgG AGG GUG AuC UUU-3′) were obtained from Hokkaido System Science. In these sequences, 2′-OMe-modified nucleotides are in lowercase, and “s” means phosphorothioate. Cholesterol was obtained from Sigma-Aldrich (St. Louis, MO, USA). Poly (ethylene glycol) (PEG; average molecular weight 2000)-dimyrystoylglycerol, *N*-hydroxysuccinimide-PEG-dystearoyl-*sn*-glycerophosphoethanolamine (NHS-PEG-DSPE), and methoxy-PEG-DSPE (MeO-PEG-DSPE) were obtained from NOF Corporation (Kanagawa, Japan). Phosphate-buffered saline (PBS) was obtained from Nacalai Tesque. ssPalmO-Phe (Product# COATSOME^®^ SS-OP) [21] was obtained from the NOF Corporation. The cRGD peptide (RGDfK: lower case means D-body amino acid) was purchased from ChemScene (Monmouth Junction, NJ, USA).

### 4.2. Cell Culture

MC38 cells were cultured in DMEM with 10% fetal bovine serum (FBS), 10 mM HEPES, 1% non-essential amino acids, 1 mM sodium pyruvate L-glutamine, and 1% penicillin and streptomycin (P/S). HUVEC and LEC cells were cultured in EGM-2 medium supplemented with 2% FBS, P/S, and attached growth factors.

### 4.3. Preparation and Characterization of LNPs

cRGD was conjugated to NHS-PEG-DSPE (RGD-PEG-lipid) via amide bonds by incubation in dimethylformamide with 1.2 equivalent of triethylamine as previously described [16,47]. siRNA-loaded LNPs were prepared by the alcohol dilution method [16,17]. ssPalmO-Phe and cholesterol in ethanol were mixed at a molar ratio of 50/50 (molar ratio) at 1500 nmol total lipid. PEG-DMG was added to the solution at 3.0 mol% to control the particle size distribution. To the lipid solution, 20 μg of siRNA in 124 μL of 20 mM malic buffer (pH 3.0) was gradually added under vigorous mixing. The mixture was sequentially diluted with 1 mL of malic buffer and then 3 mL of PBS. The resulting mixture was subjected to ultrafiltration with Amicon Ultra-14 (MWCO 100,000) twice. To modify LNP with RGD-PEG-lipid, LNP was incubated with 3.0 mol% of RGD-PEG-lipid in 10% ethanol/20 mM malic buffer solution at 45 °C for 45 min. Ethanol was removed by ultrafiltration. As a control, MeO-PEG-DSPE was incorporated into LNP instead of RGD-PEG-DSPE. The mean size and zeta potential of the prepared LNPs were determined using a Zetasizer Nano ZS ZEN3600 instrument (Malvern Instruments, Westborough, MA, USA). The encapsulation efficiency and recovery ratio of siRNA were measured by RiboGreen assay as previously described [48].

### 4.4. Flow Cytometry

LEC and HUVEC cells were washed with PBS and detached using Accutase (Innovative Cell Technologies, San Diego, CA, USA). Detached cells were incubated with anti-PE-conjugated human integrin αvβ3 antibody (Table S1) for 30 min at 4 °C. Cells were then washed with 0.5% BSA/0.1% sodium azide in PBS (FACS buffer). Ten thousand cells per sample were analyzed using a Novocyte flow cytometer (ACEA Biosciences, San Diego, CA, USA). Tumors were dissociated into single cells using mouse Tumor Dissociation Kits, and a gentleMACS™ Octo Dissociator (Miltenyi Biotec, Bergisch Gladbach, Germany). Single cells were incubated with 10 μg/mL of anti-mouse CD16/32 antibody (clone 93, Biolegend, San Diego, CA, USA) in FACS buffer for 10 min at 4 °C to block Fc receptors. After washing the cells with FACS buffer, cells were stained with fluorophore-labeled antibodies (listed in Table S1) for 30 min at 4 °C. After washing, cells were stained with 7-AAD (5 μg/mL, Biolegend, #42040) for 5 min at 25 °C to determine cell viability. Cells were analyzed using a Novocyte flow cytometer. CD8+ T cells were further isolated from single cell suspension using mouse CD8a+ T Cell Isolation Kits (Miltenyi Biotec). Isolated CD8+ T cells were stained and analyzed as described above.

### 4.5. Cellular Uptake of PEG-LNP and RGD-LNP

To investigate the cellular uptake of LNPs, HUVEC and LEC cells (1.5 × 10^5^ cells/well) were seeded on a six-well plate 24 h before LNP was added. Cells were incubated with DiO-labeled PEG-LNP or RGD-LNP at 20 μM of lipid for 2 h and then washed twice with PBS. Cells were trypsinized and centrifuged at 4 °C at 500× *g* for 5 min. The supernatant was removed with an aspirator, and FACS buffer was added to the cell pellet. The cell suspension was then analyzed on a Novocyte flow cytometer.

### 4.6. In Vitro Knockdown Studies

HUVEC cells (1.5 × 10^5^ cells/well) were seeded in 24-well plates 24 h before LNP addition. Cells were incubated with PEG-LNP at 100 nM of siPLK1 or RGD-LNPs at indicated concentrations of si*PLK1* for 24 h. Anti-human *PLK1* siRNA is used as negative control siRNA because this sequence does not have any effect on mouse *Plk1* and physiology or on immune system via RNA sensors [49]. To isolate RNA from the cells, 250 μL of TRIreagent (Molecular Research Center, Cincinnati, OH, USA) was added after washing with PBS. Total RNA was purified according to the manufacturer’s protocol. Complementary DNA was synthesized using High Capacity RNA-to-cDNA kit (ThermoFisher, Wilmington, DE, USA) at 0.5 μg RNA. The obtained cDNA was diluted 10-fold and then evaluated by quantitative PCR with THUNDERBIRD Master Mix (TOYOBO, Osaka, Japan). The expression of *PLK1* mRNA was calculated by the ΔΔCt-value method. *GAPDH* was regarded as an internal control. The primers used were *PLK1* forward; CTCCTTGATGAAGAAGATCACC, reverse; GAAGAAGTTGATCTGCACGC, *GAPDH* forward; CCTCTGACTTCAACAGCGAC, reverse; CGTTGTCATACCAGGAAATGAG as previously reported [47,50].

### 4.7. Tumor Inoculation

C57BL/6JJ mice (six weeks old, female) were purchased from Japan SLC (Shizuoka, Japan). Cancer cells were subcutaneously (s.c.) transplanted into syngeneic mice using 1 × 10^6^ cells in 100 μL Hanks’ balanced salt solution. All animal procedures were approved by the Chiba University Institutional Animal Care and Use Committee (A1-219).

### 4.8. Immunofluorescence Analysis for In Vivo Knockdown of Vegfr2

When tumor volumes reached 100–200 mm^3^, tumor-bearing mice were intravenously (i.v.) treated with RGD-LNP encapsulating either si*PLK1* as a control or si*Vegfr2*, at a dose of 40 μg siRNA/100 μL PBS/mouse. At 24 h after the administration, tumors were harvested to examine the knockdown of *Vegfr2* in MC38 tumors. The RGD-LNPs at a dose of 40 μg siRNA/100 μL PBS/mouse were i.v. administrated twice, three days apart, to tumor-bearing mice. At 24 h after the final administration, tumors were harvested to examine vascular coverage. Tumors were fixed with 4% PFA in PBS and embedded in OCT compound (Sakura Finetek, Tokyo, Japan). Tissues were sectioned (10 μm) onto glass slides (MAS coated glass, Matsunami, Osaka, Japan). The slices were then immersed in 100-fold diluted anti-mouse VEGFR2, anti-mouse CD31, and anti-mouse actin, α-Smooth muscle (αSMA) antibodies (Table S1) at room temperature for 30 min. The secondary antibodies used were goat anti-Rat IgG (H + L) Cross-Adsorbed Secondary Antibody, Alexa Fluor 647 and Goat anti-Hamster IgG (H + L) Cross-Adsorbed Secondary Antibody, Alexa Fluor 488, respectively. Images were taken using an LSM780 system (Carl Zeiss, Oberkochen, Germany). Images were then quantified using Image J software [51].

### 4.9. Tumor Inoculation

Tumor-bearing mice were randomized and treated i.p. with control IgG or aPD-1 mAb at 200 μg/mouse in 100 μL PBS and treated intravenously with RGD-LNP encapsulating si*PLK1* or si*Vegfr2* at 40 μg siRNA/mouse in 100 μL PBS on days 5, 8, and 12 (post-tumor inoculation). Tumor volume was calculated using the formula 1/2 × a × b^2^, where a and b represent the largest and smallest tumor diameters, respectively. Tumor volumes of 30% or less than that of tumors treated with control IgG were regarded as efficiently suppressed.

### 4.10. Statistical Analysis

All data are presented as the mean value ± S.E. Pair-wise comparisons of subgroups were made using Student’s *t*-tests with Welch’s correction. Comparisons between multiple treatments were made using one-way analysis of variance (ANOVA), followed by an appropriate post-hoc test, and chi-squared tests followed by adjusted standardized residual analysis. *P* values (both sides) were considered significant if *p* was less than 0.05. Statistical analyses were performed using GraphPad Prism 5.0 (GraphPad Software, San Diego, CA, USA). An interaction index of combination therapy versus monotherapy of the fixed-dose drug combination was assessed according to the methods described in a previous report [22]. If the 95% CI was less than zero, the drug combination was considered to be supra-additive; if it contained zero, the drug combination was considered to be additive; otherwise, the drug combination was considered to be sub-additive.

## 5. Conclusions

In this study, we demonstrated successful siRNA delivery to TECs in xenograft tumors using RGD-modified LNP composed of ssPalmO-Phe. The combination of aPD-1 mAb and VEGFR2 knockdown decreased the number of TILs in tumors via vascular normalization and suppressed tumor growth. Further studies are required to test whether this combination strategy could be applied to other types of cancer therapies, but these results indicate that this delivery system implements a combination strategy which can improve the therapeutic outcome of ICIs.

## Figures and Tables

**Table 1:** Characterization of LNPs.

Formulation	Diameter (nm)	PDI	ξ-Potential (mV)	EE (%)	RR (%)
PEG-LNP	157 ± 4	0.21 ± 0.03	−9 ± 4	95.2 ± 1.6	67.5 ± 1.6
RGD-LNP	167 ± 6	0.22 ± 0.01	−11 ± 4	91.9 ± 2.6	58.1 ± 10.9

PEG-LNP, PEGylated lipid nanoparticle; RGD-LNP, RGD modified lipid nanoparticle; EE, encapsulation efficiency; RR, recovery rate; LNP, lipid nanoparticle; PDI, polydispersity index; Mean ± SE, *n* = 3.

## Supplementary Materials

The following are available online at https://www.mdpi.com/2072-6694/12/12/3630/s1, Table S1: List of antibodies used in the study.
